# Acoustic Transmitted Decellularized Fish Bladder for Tympanic Membrane Regeneration

**DOI:** 10.34133/research.0596

**Published:** 2025-02-05

**Authors:** Hong Chen, Hui Zhang, Guangjie Zhu, Long Cao, Chenjie Yu, Maoli Duan, Xiaoyun Qian, Xia Gao, Yuanjin Zhao

**Affiliations:** ^1^ Department of Otolaryngology Head and Neck Surgery, Nanjing Drum Tower Hospital, Research Institute of Otolaryngology, Jiangsu Provincial Key Medical Discipline, Nanjing University Medical School, Nanjing 210008, China.; ^2^ School of Life Sciences and Technology, Southeast University, Nanjing 210096, China.; ^3^ Institute of Translational Medicine, Medical College, Yangzhou University, Yangzhou 225001, Jiangsu, China.; ^4^ Department of Otolaryngology Head and Neck Surgery & Audiology and Neurotology, Karolinska University Hospital, Karolinska Institute, 171 76 Stockholm, Sweden.

## Abstract

Developing advanced tissue-engineered membranes with biocompatibility, suitable mechanical qualities, and anti-fibrotic and anti-inflammatory actions is important for tympanic membrane (TM) repair. Here, we present a novel acoustically transmitted decellularized fish swim bladder (DFB) loaded with mesenchymal stem cells (DFB@MSCs) for TM perforation (TMP) repair. The DFB scaffolds are obtained by removing the cellular components from the original FB, which retains the collagen composition that favors cell proliferation. Benefitting from their spatially porous structures and excellent mechanical properties, the DFB scaffolds can provide a suitable microenvironment and mechanical support for cell growth and tissue regeneration. In addition, by loading mesenchymal stem cells on the DFB scaffolds, the resultant DFB@MSCs system exhibits remarkable anti-fibrotic and anti-inflammatory effects, together with the ability to promote cell migration and angiogenesis. In vivo experiments confirm that the prepared DFB@MSCs scaffolds can not only alleviate inflammatory response caused by TMP but also promote new vessel formation, TM repair, and hearing improvement. These features indicate that our proposed DFB@MSCs stent is a prospective tool for the clinical repair of TM.

## Introduction

The translucent tissue known as the tympanic membrane (TM) divides the middle ear from the outer ear [1–3]. It is essential for both middle ear protection and sound perception. In daily life, various factors such as inappropriate ear use, middle ear inflammation, pneumatic pressure injuries, and postoperative otologic complications can cause TM perforation (TMP), resulting in hearing loss and recurrent middle ear infections [2,4,5]. Clinically, autografts like autologous temporal fascia, cartilaginous membrane of the ear screen, and adipose tissue have been exploited to repair TMP [6,7]. Although with some therapeutic outcomes, these traditional grafts have unavoidable postoperative complications, such as anesthesia risk, incision hematoma, pain, infection, and keloid scarring. Recently, tissue-engineered membranes with high availability and biocompatibility have been satisfactory candidates for the repair of TMP [8–11]. Both synthetic polymers (like silicone rubber membrane and polytetrafluoroethylene membrane) and natural polymers (like gelatin, silk fibroin chitosan, alginate, and hyaluronic acid) have been explored for engineering scaffold construction [10,12–14]. Among them, benefiting from their charming intrinsic properties, naturally original materials have been confirmed with extension applications over synthetic materials. Nevertheless, most of these natural polymer-derived artificial membranes generally need to be reproduced through complex procedures, and their products showed difficulty in balancing elasticity and mechanical strength. Therefore, new tissue-engineered membrane based on natural materials for TM repair is still expected.

**Fig. 1.**
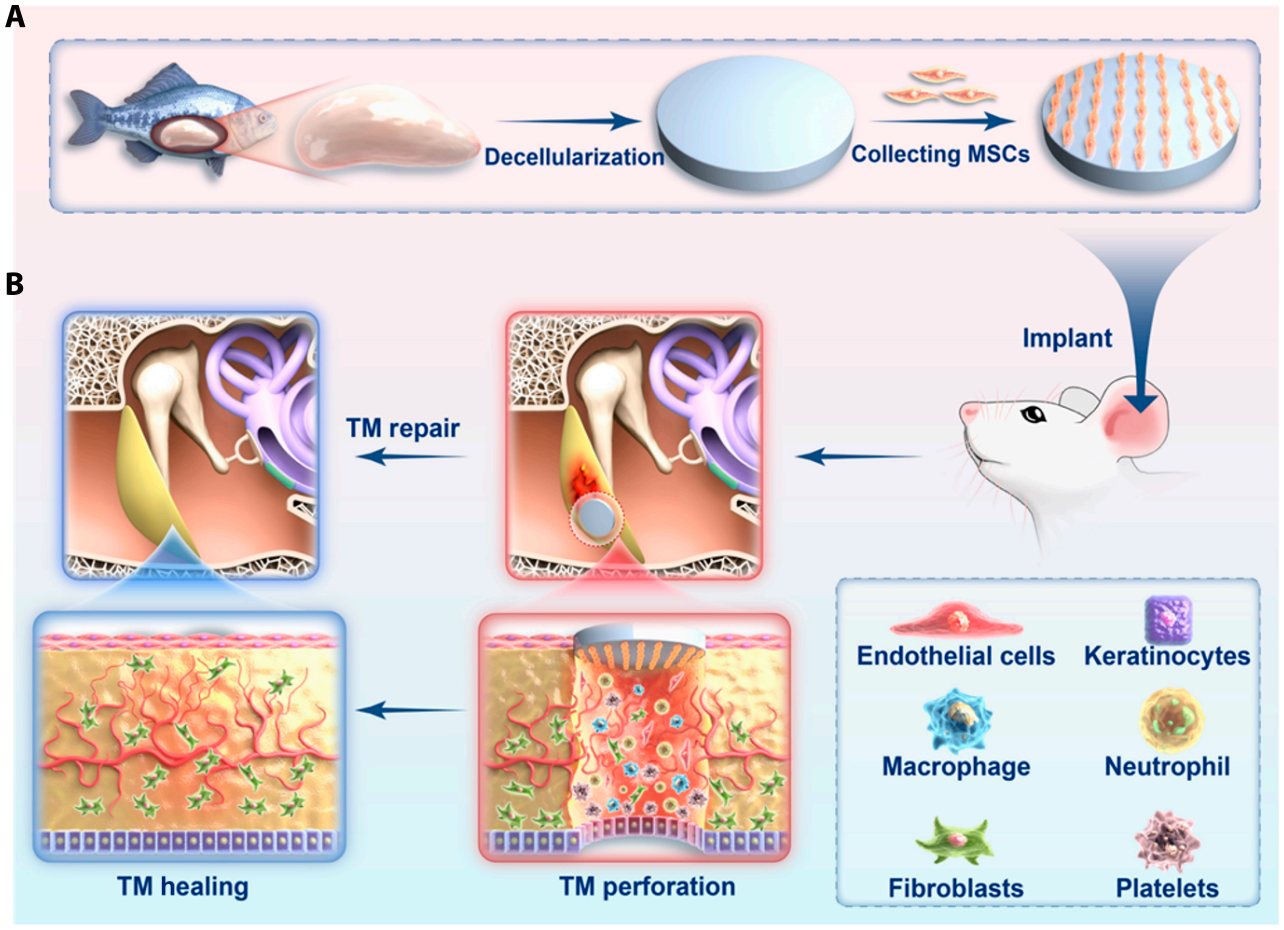
Schematic diagram of DFB@MSCs with desired features for TM repair. (A) Preparation process of DFB@MSCs. (B) TM repair with DFB@MSCs.

Herein, we propose a novel mesenchymal stem cell (MSC)-loaded acoustic transmitted decellularized fish swim bladder (DFB@MSCs) with desired features for TM repair, as schemed in Fig. 1. Fish swim bladder (FB), an organ that helps fish float freely in water and is abundant, inexpensive, antigenic, and hypoallergenic, is a potential animal-derived collagen biomaterial generated from naturally occurring aquatic materials [15–18]. As the FB is derived from aquatic organisms and there are no reports of zoonotic transmission of fish viruses, it is less contaminated and safer than terrestrial organisms, which indicated that its use as a material for TM repair has potential application prospects [19,20]. Additionally, MSCs are pluripotent stem cells that possess the capacity to develop in several directions and self-renew. Following transplantation, they can transform into fibroblast resident cells, vascular endothelial cells, and other local tissues [21–26]. Besides, it has also been demonstrated that MSCs increase regulatory T cells, release anti-inflammatory cytokines, and inhibit costimulatory molecules on antigen-presenting cells [27–33]. The significance of MSCs in TM repair is that they can promote TM healing by being influenced by the microenvironment of the ear to differentiate into fibroblasts. Thus, we conceptualized that combining FB with MSCs will develop a new artificial TM system.

In this paper, we constructed the desired FB-derived grafts through decellularizing treatment and employed the resultant scaffolds for MSC transplant and TMP repair. With the treatment of sodium dodecyl sulfate (SDS) and Triton X-100, the cellular constituents within raw FB were entirely removed while the original collagen components were preserved, which contributed to cell survival and proliferation. Besides, the obtained DFB scaffolds possessed spatially porous structures and excellent mechanical performances, thereby providing a suitable microenvironment and physical support for cell growth and tissue regeneration. In addition, MSCs were implanted into the DFB scaffolds to endow them with remarkable anti-fibrotic and anti-inflammatory properties. In vitro studies revealed that DFB@MSCs could not only reverse the differentiation of fibroblasts to myofibroblasts but also effectively promote cell migration and angiogenesis. After implanting these DFB@MSCs scaffolds into the TM damage site, it was demonstrated that the scaffold attenuated the inflammatory response caused by TMP and facilitated microvascular regeneration, thus improving TM repair outcomes. These results suggested that the DFB@MSCs scaffolds can be a promising candidate for clinically repairing TMP.

## Results and Discussion

### Preparation and characterization of FB scaffolds

**Fig. 2.**
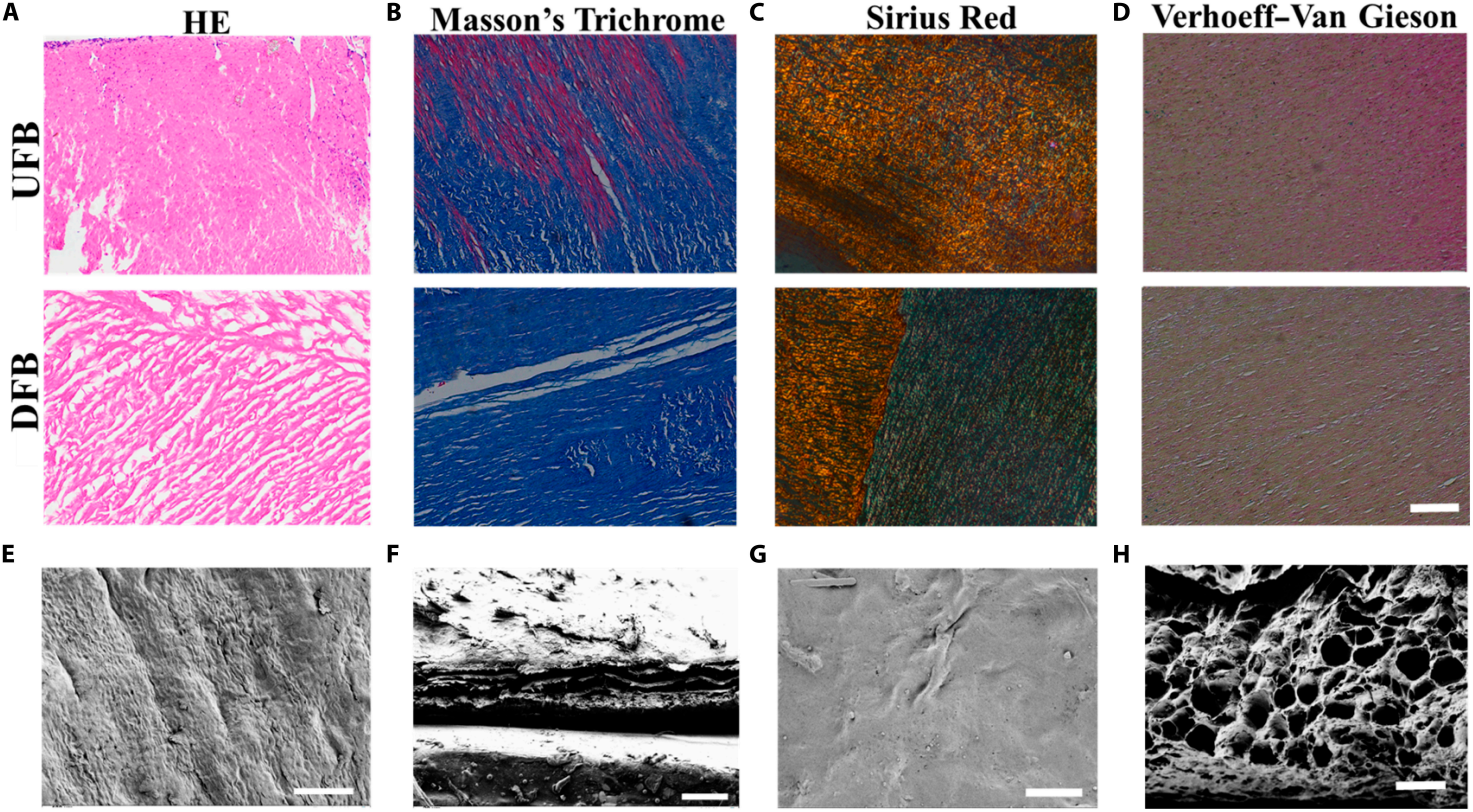
Characterization of UFB and DFB scaffolds. (A) HE staining images of UFB and DFB Scaffolds. (B) Masson’s trichrome staining images of UFB and DFB scaffolds. (C) Sirius Red staining images of UFB and DFB scaffolds. (D) Verhoeff–Van Gieson staining images of UFB and DFB scaffolds. (E) SEM picture showing the UFB scaffold’s surface. (F) SEM picture showing UFB scaffold’s cross-section. (G) SEM picture of DFB scaffold’s surface. (H) SEM picture of DFB scaffold’s cross section. [Scale bars: 200 μm (D), 100 μm (E and G), and 50 μm (F and H).]

In a typical experiment, we collected native FB (UFB) from the market, whose surface showed blood vessels and fatty tissues (Fig. S1A and B). Then, the UFB was decellularized using SDS and Triton X-100 and lyophilized to obtain DFB (Fig. S2A and B). As shown by hematoxylin-eosin (HE) staining in Fig. 2A, no nuclei were observed inside the FB after decellularization, which was also verified by quantitative DNA detection analysis (Fig. S2C). These results revealed that cells and cellular debris were removed from the FB. In addition, Masson’s trichrome, Sirius Red, and Verhoeff–Van Gieson staining images showed no significant changes in the fibrous structures, indicating that the decellularization treatment could retain the extracellular matrix structure and components of the UFB (Fig. 2B to D). In order to systematically observe the surface microstructures of UFB and DFB, scanning electron microscopy (SEM) analysis was displayed. As exhibited in Fig. 2E to H, the UFB surface had compact surface and interior structures, whereas the DFB showed a flat surface and porous network structure conducive to cell adhesion and growth. The high water content of scaffolds ensures the transfer of essential nutrients as well as metabolites required for cell survival. Studying scaffold water retention is therefore essential. In this study, the water absorption of each group was compared. As shown in Fig. S2D, there is no difference between DFB and UFB, with a water uptake capacity 2 times the original.

### Mechanical properties of FB scaffolds

**Fig. 3.**
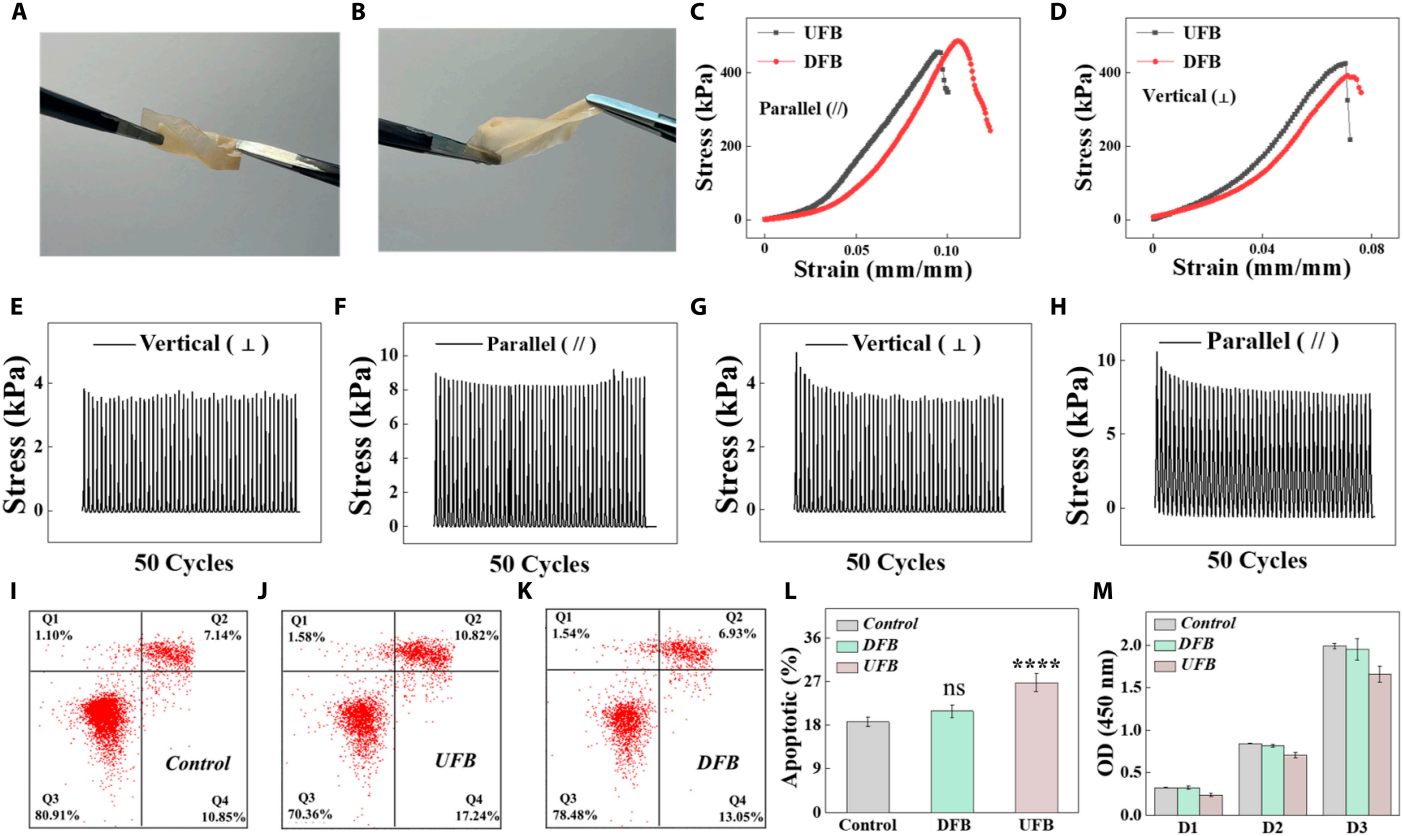
The mechanical properties and biocompatibility of FB scaffolds. (A) Representative images of DFB film in the torsional deformation test. (B) Representative images of DFB film in the stretching test. (C and D) Tensile stress–strain curves of UFB and DFB scaffolds in parallel (C) and vertical (D) directions. (E and F) Cyclic tensile test of UFB scaffolds in vertical (E) and parallel (F) directions. (G and H) Cyclic tensile test of DFB scaffolds in vertical (G) and parallel (H) directions. (I to K) Analysis of House Ear Institute–Organ of Corti 1 (HEI-OC1) cells using flow cytometry following a 24-h growth period on various substrates. (L) Apoptosis statistics according to flow cytometry result (*n* = 3). (M) HEI-OC1 cells’ Cell Counting Kit-8 (CCK-8) findings during a 3-day incubation period with various substrates. (****P < 0.0001; ns, denotes no discernible variation from the control group.)

To determine the applicability of FB materials as repair scaffolds for TMP, we evaluated their mechanical properties. As shown in Fig. 3A and B, DFB film showed favorable flexibility during torsional deformation (Fig. 3A) and stretching tests (Fig. 3B). Besides, due to the inhomogeneous microstructures, the anisotropic mechanical properties of UFB and DFB scaffolds loaded horizontally and vertically were investigated. It was found that there was no significant difference in the mechanical properties of UFB and DFB, both vertically and horizontally (Fig. 3C and D). After 50 repetitive tensile cycles in the horizontal and vertical directions, there was no stress loss in both UFB and DFB scaffolds, indicating good elasticity of FB even after decellularization (Fig. 3E to H). Overall, DFB with excellent mechanical performance could provide satisfactory physical support for TM reconstruction following in vivo implantation.

### In vitro biocompatibility evaluation of FB scaffolds

To investigate the biocompatibility of FB scaffolds, HEI-OC1 cells were cultured with the supernatant of FB scaffolds and an apoptosis assay was carried out using flow cytometry, with the cells in the control group being treated with pure culture media. The findings revealed that the cell apoptosis rates in the control, DFB, and UFB groups were 18.765%, 20.915%, and 26.77%, respectively, after 24 h of cultivation (Fig. 3I to L). In comparison to the control and DFB groups, the UFB group exhibited a higher rate of cell death. Additionally, the cell viability in different groups was assessed by CCK-8 detection, which yielded consistent results with the flow cytometry analysis, as shown in Fig. 3M. To visually analyze the cell morphology of HEI-OC1 cells, we performed live/dead staining on days 1, 2, and 3. As a result, HEI-OC1 cells in all groups displayed normal morphology; however, the UFB group had more dead cells with red fluorescent signals than the control and DFB groups (Fig. S3). In order to further validate the biocompatibility of DFB scaffolds, we performed live/dead experiments by directly culturing HEI-OCI cells and MSCs on the scaffolds for 48 h. The findings revealed that the HEI-OC1 cells and MSCs grew uniformly and morphologically on DFB scaffolds with minimal dead cells (Fig. S4). Moreover, immunofluorescence staining was conducted to examine the morphological characteristics of MSCs in different groups. It was found that MSCs grew uniformly with good morphology on the DFB scaffolds, and cell numbers gradually increased (Fig. S5). These results confirmed that the DFB scaffolds were highly biocompatible, contributing to the survival and proliferation of HEI-OC1 cells and MSCs.

### In vitro antifibrosis and anti-inflammatory studies of DFB@MSCs scaffolds

**Fig. 4.**
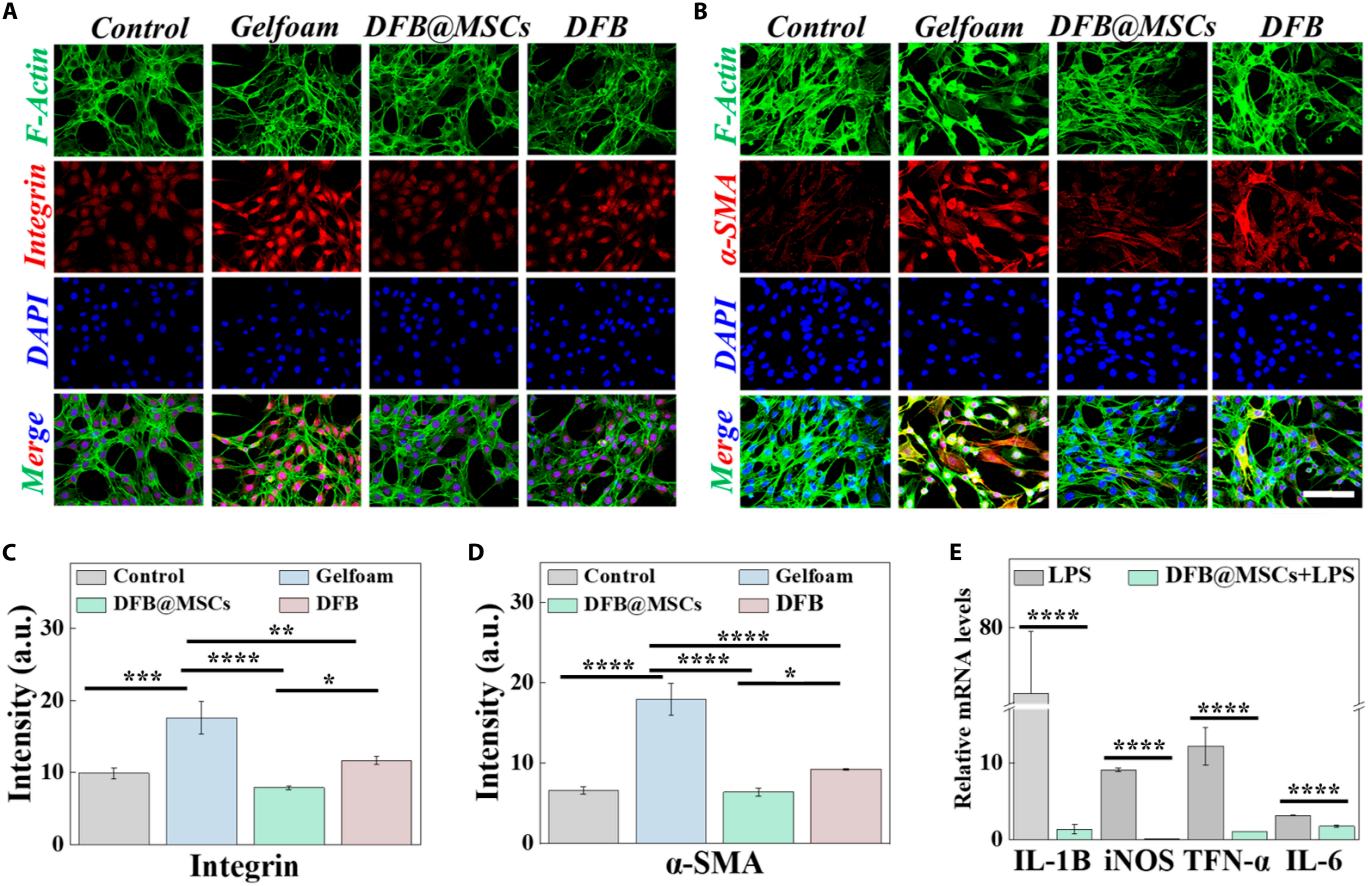
Evaluation of antifibrosis and anti-inflammatory properties of DFB@MSCs. (A and B) Immunofluorescent staining images of integrin (A) and α-SMA (B) of MSCs in various groups. Scale bar: 100 μm. (C) The integrin signal’s fluorescence intensity (*n* = 3). (D) Fluorescence intensity of the α-SMA signal. (E) Relative mRNA level for IL-6, IL-1β, iNOS, and TNF-𝛼 in epithelial cells with different treatments (*P < 0.05, **P < 0.01, ***P < 0.001, ****P < 0.0001).

It is well known that posttraumatic scar adhesion contributes to inadequate tissue regeneration; therefore, while constructing tissue replacement scaffolds, it is essential to preserve cell viability and encourage tissue proliferation without aggravating scar formation. We evaluated the impact of DFB@MSCs on fibroblasts using α-smooth muscle actin (α-SMA) and integrin immunofluorescence labeling. After 2 days of cultivation, fibroblasts showed normal cytomorphology, while fibroblasts in the Gelfoam group displayed the strongest α-SMA and integrin signals, as seen in Fig. 4A and B. Statistics on fluorescence intensities revealed that integrin signals of the Gelfoam group (17.608 ± 2.247) were substantially greater than the control group (9.911 ± 0.780), the DFB@MSCs group (7.902 ± 0.261), and the DFB group (11.692 ± 0.577) (Fig. 4C). Similarly, fluorescence intensities of the α-SMA signal of the Gelfoam group (17.975 ± 1.957) were notably greater than the control group (6.609 ± 0.445), the DFB@MSCs group (6.414 ± 0.488), and the DFB group (8.226 ± 0.127) (Fig. 4D). According to these results, compared with the commercial product Gelfoam, DFB@MSCs scaffolds could effectively reverse the transdifferentiation of fibroblasts into myofibroblasts due to the integration of MSCs.

In addition, we examined the anti-inflammatory properties of DFB@MSCs by real-time qPCR at the mRNA level. Epithelial cells were cocultured with different mediators for 24 h under inflammatory stimuli induced by LPS. The results showed that inflammation-related genes, including interleukin (IL)-1β, IL-6, inducible nitric oxide synthase (iNOS), and tumor necrosis factor (TNF-𝛼), were down-regulated in the DFB@MSCs group compared with those of only the LPS-stimulated group (Fig. 4E). In particular, there was no difference in inflammatory markers in the MSC and DFB groups compared to the control group, which revealed that neither MSC nor DFB scaffolds could cause inflammatory responses (Fig. S6). These findings suggest that combining MSCs with DFB scaffolds displayed remarkable anti-inflammatory properties and may serve as a potential anti-inflammatory scaffold for TMP.

### Evaluation of cell migration-promoting and angiogenic effects of DFB@MSCs scaffolds

**Fig. 5.**
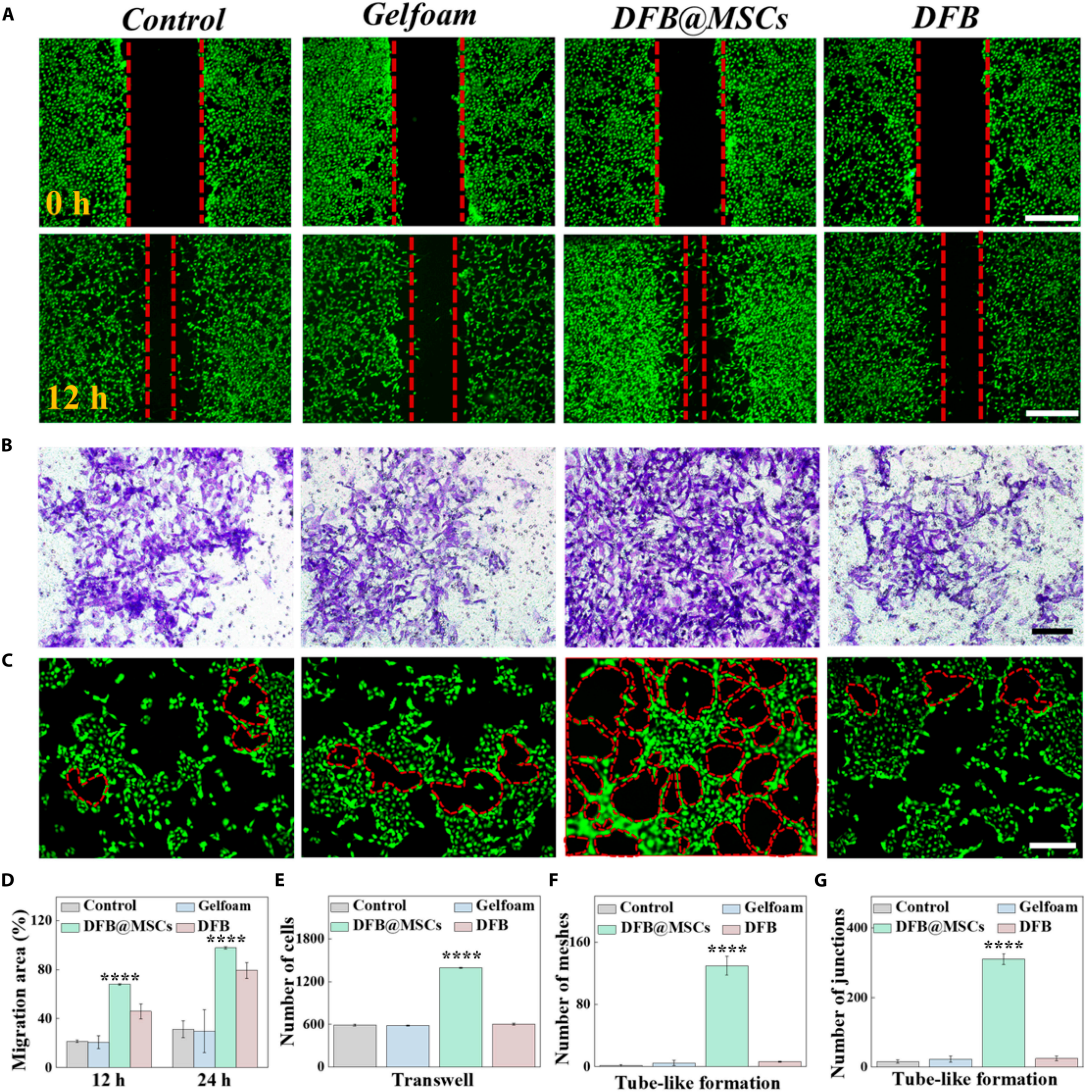
Study of cell migration and angiogenesis. (A) Scratch test results of epithelial cells in different groups at 0 and 12 h. Scale bars: 500 μm. (B) Vertical migration crystal violet photos of epithelial cells in different groups. Scale bar: 100 μm. (C) Fluorescent staining photos of HUVECs in different groups. Scale bar: 100 μm. (D) Migration areas in different groups at 12 h. (E) Counts of transwell migrating cells in different groups. (F and G) Quantitative assessment of angiogenesis experiments (****P < 0.0001).

Cell migration and revascularization are crucial parts of the TM repair and reconstruction process. Thus, we performed migration and angiogenesis assays to investigate these abilities of DFB@MSCs scaffolds. The DFB@MSCs group exhibited a considerably greater migration rate of fibroblastic epithelial cells at 12 h in the cellular scratch test compared to the other 3 groups (Fig. 5A and D). Regarding the transwell migration assay, it was observed from crystal violet staining images that the number of cells passing through the transwell insert in the DFB@MSCs group was more than that in the other groups (Fig. 5B and E). There was no significant difference between the control, Gelfoam, and DFB groups. The above results indicated that DFB@MSCs scaffolds effectively accelerated the migration of epithelial cells. Furthermore, we explored human umbilical vein endothelial cells’ (HUVECs’) tube-forming capacity grown within various conditions. As exhibited in Fig. 5C, HUVECs of the DFB@MSCs group showed more tubular structures than the other groups, confirming the superior proangiogenic capability of DFB@MSCs scaffolds (Fig. 5F and G).

### In vivo assessment of DFB@MSCs scaffolds for TM regeneration

**Fig. 6.**
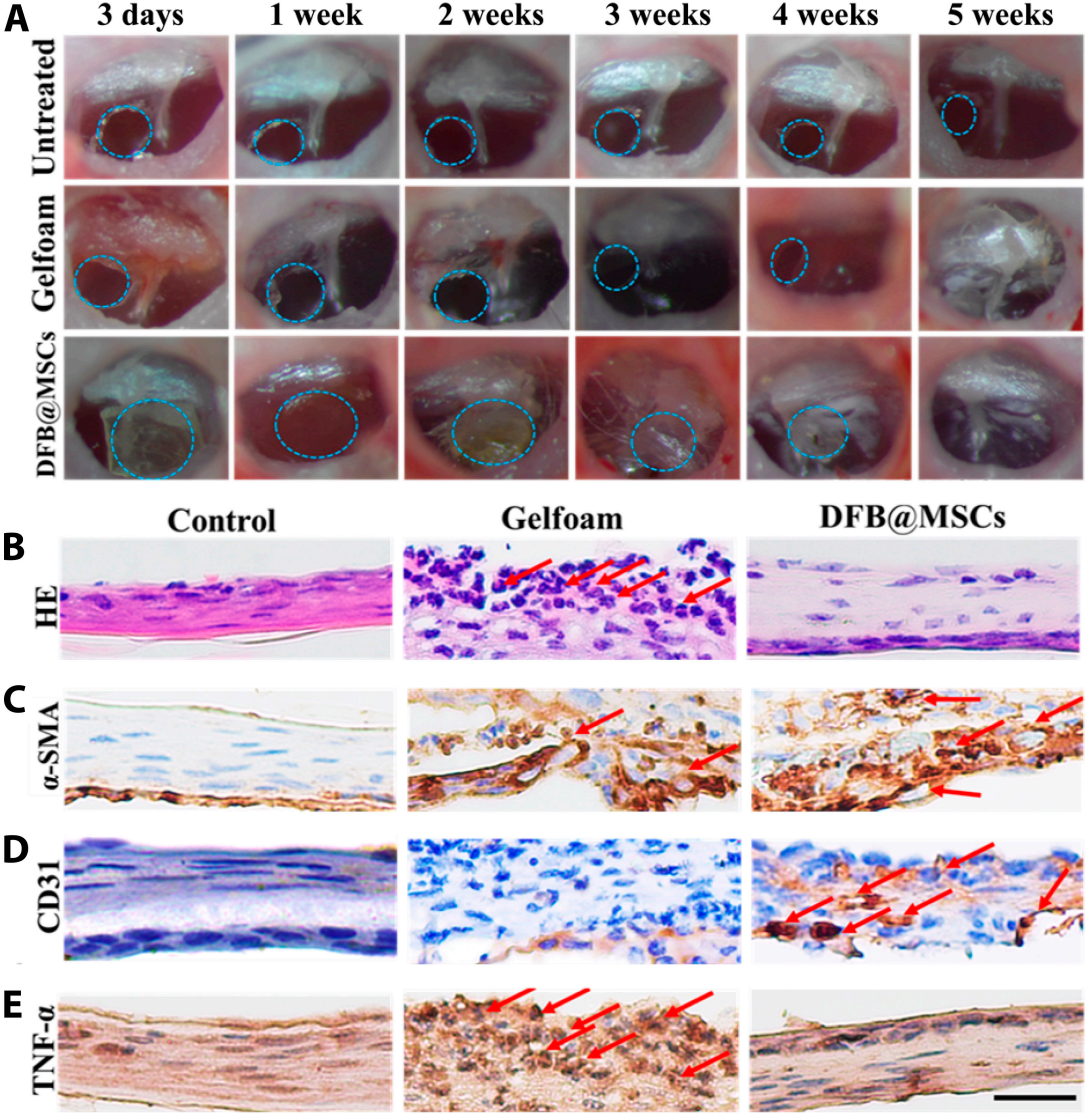
Evaluation of TM regeneration. (A) Images of TM that are representative of several groups. (B) HE staining photos of TM in various groups 5 weeks postsurgery. (C and D) α-SMA (C) and CD31 (D) immunohistochemical staining pictures of TM following 5 weeks of surgery in various groups. (E) TNF-α immunohistochemical staining photos of several groups of TM. (F to H) Statistical analysis of inflammatory factors in the middle ear. Scale bar: 20 μm.

Animal experiments were conducted to ascertain whether the DFB@MSCs scaffold is appropriate for wound healing in chronic TM regeneration. In detail, mice were used to create chronic TMP models under a surgical microscope. The animals were then arbitrarily split into different groups: the control group without any treatment, the untreated group without scaffold implantation after surgery, the Gelfoam group that had a Gelfoam scaffold implanted, and the DFB@MSCs group with DFB@MSCs scaffold implantation, with 5 mice in each group. At various time intervals, the TM healing situation was observed and recorded (Fig. 6A). It was found that the mice in the untreated group showed an unobvious repair effect after 5 weeks. Besides, all of the chronic perforation models in the Gelfoam group reported the presence of more calcified areas on TM. In contrast, the DFB@MSCs group had a more complete healing at week 5, with fewer calcified areas than the Gelfoam group. To assess the structural characteristics of TMs reconstructed with DFB@MSCs scaffolds, HE staining images of regenerated TM were obtained after 5 weeks of surgery (Fig. 6B). It was observed that the regenerated TM in the DFB@MSCs group recovered the 3-layered structure with complex squamous epithelium, fibrous layer, and mucous membrane layer. TM samples from the Gelfoam group, on the other hand, displayed a significant number of inflammatory cells (shown by arrows) and lacked a distinct 3-layer pattern. These findings revealed that DFB@MSCs increased the healing rate and accelerated the healing pathway compared to the commercial Gelfoam product. Of note, HE staining images of major organs showed no abnormalities after 5 weeks of treatment, indicating that DFB@MSCs were not toxic to mice (Fig. S7).

Angiogenesis is another necessary indicator of the process of TM remodeling. Therefore, we performed immunohistochemical staining for α-SMA and CD31 to determine the neovascularization of regenerated TM with different treatments. The findings revealed notable differences in the 3 groups’ vascular structure densities (Fig. 6C and D). In the control group, there were almost no positive signals for α-SMA and CD31, suggesting few vascular structures at the regenerated TM site. In contrast, higher levels of α-SMA and CD31 expression were observed in the DFB@MSCs group over both the control group and the Gelfoam group, which indicated a positive effect of DFB@MSCs scaffold on the revascularization after TMP.

Chronic inflammation is a key factor hindering tissue regeneration after TMP. To assess the inflammation and structural characteristics of regenerated TM following the implantation of DFB@MSCs scaffolds, immunohistochemical staining images were obtained 5 weeks after surgery. As illustrated in Fig. 6E, the DFB@MSCs group had lower levels of TNF-𝛼 (brown-stained irregular cells) than the Gelfoam group.

### In vivo evaluation of the hearing level and the hair cell morphology after scaffold implantation

**Fig. 7.**
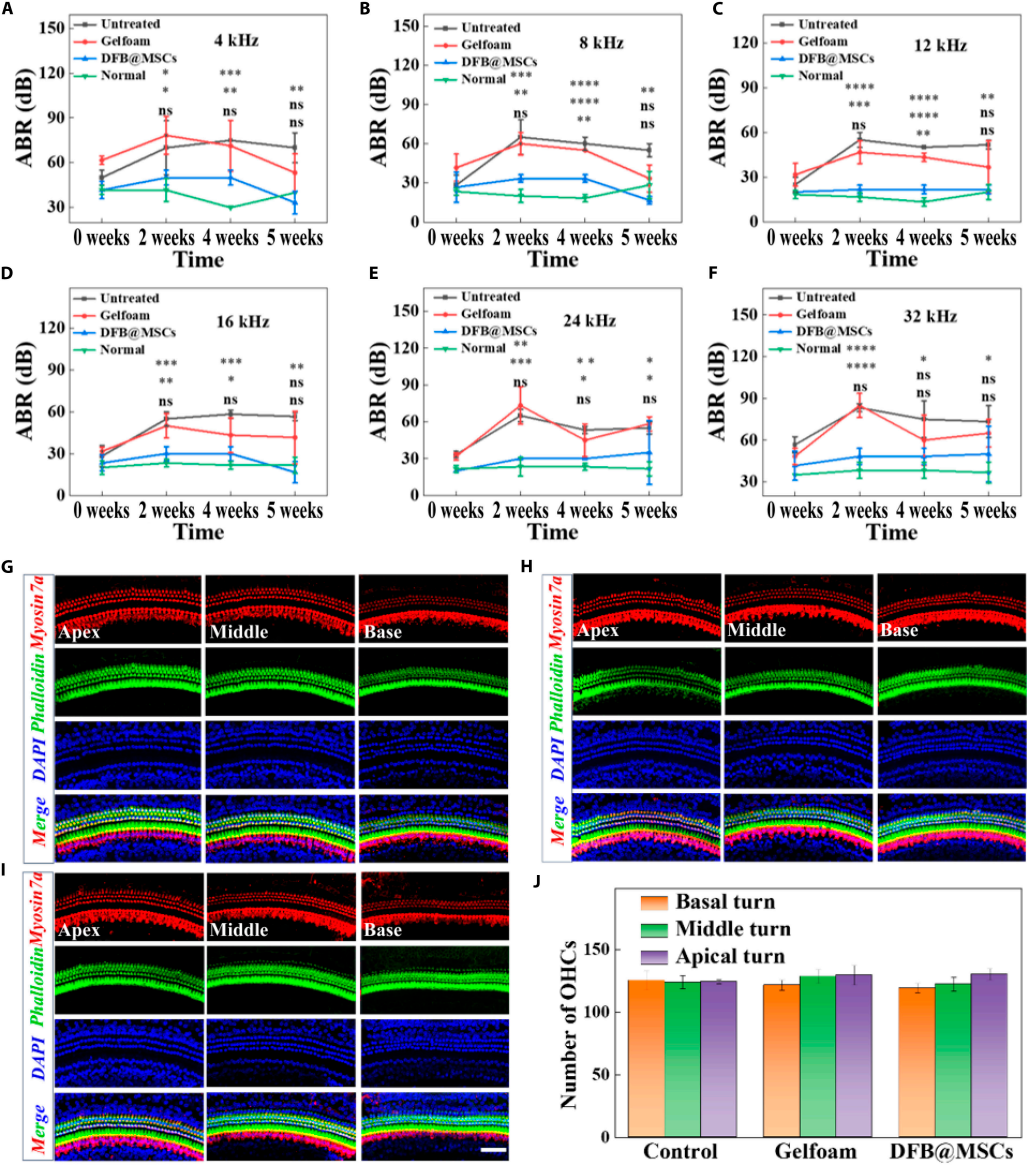
Hearing level and hair cell morphology of mice with different treatments. (A to F) ABR thresholds of mice in different groups within 5 weeks (*n* = 3, *P < 0.05, **P < 0.01, ***P < 0.001, ****P < 0.0001). (G to I) Immunofluorescent staining images of each group’s cochlear basement membrane of mice in each group. (J) Statistical evaluation of each group’s OHCs. Scale bar: 50 μm.

Moreover, as TMP can damage the hearing level, the auditory brainstem response (ABR) test threshold was used as an objective parameter reflecting hearing level to evaluate TM repair. The animals in the DFB@MSCs group did not have significant hearing loss during the TMP healing process, as shown in Fig. 7A to F. By the fifth week, their hearing threshold had returned to a healthy level comparable to that of normal mice. By contrast, the mice in the Gelfoam group and untreated group exhibited a noticeable decrease in hearing after 2 weeks of surgery and did not recover to normal hearing levels by the fifth week. In the process of sound transmission, the outer hair cells (OHCs) of the cochlea are the amplifiers of sound and are also the most vulnerable parts. Ototoxic drugs, noise, aging, and other factors can cause damage to the external hair cells. When OHCs are damaged, they lose the ability to regenerate their own hair cells, which can lead to severe hearing loss. The basilar membrane hair cells of mice from different treatment groups were collected, stained, and statistically analyzed. As illustrated in Fig. 7G to I, there was minimal hair cell loss in the basilar membrane in all groups. Besides, we counted the number of OHCs based on immunofluorescent staining images, and the statistical analysis showed no significant difference. These findings further demonstrate the TMP repair-promoting ability and high biocompatibility of the DFB@MSCs scaffolds in vivo.

Additionally, the basilar membrane hair cells of mice from different treatment groups were collected, stained, and statistically analyzed. As illustrated in Fig. 7G to I, there was minimal hair cells loss in the basilar membrane in all groups. Besides, we counted the number of OHCs based on immunofluorescent staining images, and the statistical analysis showed no significant difference. These findings further demonstrate the TMP repair-promoting ability and high biocompatibility of the DFB@MSCs scaffolds.

## Conclusion

In conclusion, we presented acoustic-transmitted DFB@MSC scaffolds for TM regeneration. After decellularization, the immunogenic components within the natural FBs were removed entirely, while the original collagen components were well retained. The obtained DFB scaffolds exhibited a spatially porous structure and excellent mechanical properties conducive to cell outgrowth and tissue regeneration. Moreover, the integration of MSCs endowed the DFB scaffolds with optimized properties, which could reverse the differentiation of fibroblasts to myofibroblasts, attenuate inflammatory responses, and promote cell migration and angiogenesis. The implantation of resultant DFB@MSCs scaffolds into the TMP site effectively reduced the inflammation and facilitated neovascularization, thus improving TM repair. Additionally, the DFB@MSCs scaffolds could alleviate the decrease in hearing levels by TMP and restore it to normal. These results confirmed the potential of DFB@MSCs scaffolds for TM regeneration.

## Methods

### Decellularization of FB

After collecting the anterior portion of the FBs, the fat and vascular tissues were carefully removed from their surfaces and the FBs were rinsed 3 times in sterile phosphate buffer solution (PBS) for 10 min each time. After that, the FBs were placed in 1 wt% SDS and shaken at 4 °C for 72 h, with solution changes every 12 h. Afterwards, the FBs were treated by 1 vol% Triton X-100 at 4 °C for 72 h, during which the liquid was changed every 12 h, finally obtaining DFB scaffolds. Next, the DFB scaffolds were washed several times in sterile PBS until there was no foam in the solution. Finally, the cleaned DFB scaffolds were placed in sterile PBS and stored at 4 °C.

### Mechanical properties test

After being submerged in PBS for 20 min, the UFB and DFB scaffolds were divided into 5 cm × 4 cm pieces. Then, these scaffolds were fastened to a mechanical testing device, and their maximum tensile force was recorded when the material was broken. Besides, 50 cycles of tensile testing were conducted at a compression rate of 0.2 mm/s. Both parallel (//) and perpendicular (⊥) orientations were used to examine the scaffolds’ tensile strength. At least 3 samples were tested in each group.

### Biocompatibility assay

For a whole day, aseptic UFB and DFB scaffolds were immersed in 1 ml of cell culture media at 37 °C. HEI-OC1 cells were seeded and split into the control, UFB, and DFB groups, wherein the cells in the UFB and DFB groups were treated with the corresponding supernatant while the cells in the control group were grown in standard media. On the one hand, the cells were stained with a Live/Dead Staining Kit for 30 min on days 1, 2, and 3. On the other hand, cell proliferation was measured by the CCK-8 test.

### Flow cytometry

The cells were split into 3 groups: the control, UFB, and DFB groups. Following a 24-h growth period in various media, cells were harvested and dyed with the Annexin V-FITC Kit, and then flow cytometry was used to identify the results.

### RNA extraction

After being stimulated with LPS for 1 day, epithelial cells were cocultured in various media. One day later, RNA was extracted from different subgroups of cells using an RNA extraction kit. Utilizing the Nanodrop 2000c, the concentration and purity of RNA were assessed. After that, RNA was changed into cDNA. Lastly, the expression of mRNA is quantified, with GAPDH serving as the reference (Table S1).

### Wound-scratch assay

Firstly, 3 horizontal lines of equal width were drawn on the bottom of the 6-well plate as a subsequent observation mark. Then, the epithelial cells at a concentration of 1×10^5^/ml were introduced into the 6-well culture plate. A 200-μl pipette tip was employed to create a scratch perpendicular to the 6-well plate and the previously marked lines along the center line of the wells, thereby forming a “wound”. Next, the original medium was removed, and 1 ml of corresponding supernatant was added. After 12 h of incubation, the epithelial cells were labeled with calcein AM and cell migration was observed and photographed using a microscope. The healing degree of the scratched area was measured using ImageJ, and the cell migration rate was further calculated.

### Transwell assay

Transwell Permeable Supports (8.0 μm) were used. The epithelial cells were digested and resuspended in a serum-free medium at a concentration of 5 × 10^5^/ml. Extraction solution (500 μl) was added to the lower chamber and 200 μl of cell resuspension solution was added to the upper chamber. After 24 h of culture, the chambers were taken out, and the medium was washed away with PBS and stained with crystal violet for 10 min. Then, the surface was washed clean of crystal violet with tap water, and the cells on the inoculated side in the upper chamber were wiped clean with a cotton swab. The pictures were captured of the noncellular inoculated side under a microscope.

### Tube formation assay

First, the Matrigel was removed from the −20 °C refrigerator and placed in the 4 °C freezer until it was fully thawed. Then, the Matrigel was mixed to a homogeneous state using a precooled gun tip. Next, 100 μl of Matrigel was added to each well of the precooled 48-well plate. The 48-well plate was transferred to a cell culture incubator and incubated at 37 °C for 30 min. Subsequently, 2×10^4^ HUVECs were inoculated into each well and incubated with 300 μl of extraction solution. Finally, after 24 h, HUVECs were labeled with calcein AM and tube formation on each well was examined under an electron microscope. The results were statistically analyzed by ImageJ.

### Fibroblast immunofluorescence staining

After being cultured in different media for 2 days, fibroblasts were fixed in a 4 wt% perfluoroalkoxy (PFA) solution for 1 h and washed 3 times by adding 1 vol% PBST. The primary antibodies were added after blocking for an hour, and then the matching secondary antibodies, DAPI (4′,6-diamidino-2′-phenylindole), and phalloidin were added the next day and incubated for an additional hour. After washing 3 times, the cell slices were sealed and observed by confocal microscope.

### Animal experiment

The Animal Ethics Committee of Nanjing Drum Tower Hospital authorized all animal experiments using mice (2023AE01003). Forty-day mice were utilized for TMP model construction. After anesthesia and local disinfection, the perforated TM was exposed under a microscope. For TMP, the edges of the perforation were scraped with a crochet hook to create a fresh wound. Then, the perforated TM was repaired using an appropriately sized scaffold. To immobilize the graft in place as well as to prevent stenosis of the external ear canal, the mouse ear canal was filled with iodized gauze.

### Histologic analysis

The TM tissues were collected and stored in 4 wt% PFA for fixation before paraffin embedding. Then, HE and immunohistochemical staining of CD31, α-SMA, and TNF-α were carried out to evaluate the TM repair performance.

### ABR test

The anesthetized mice were placed on the Tucker-Davis Technologies workstation, and the electrodes were inserted into the mice in a certain sequence. Frequencies of 4 to 32 kHz were tested, and the BioSigin application was started on a computer. Each frequency was analyzed beginning at 90 dB. Origin software was used to compile and evaluate all of the captured data.

### DNA concentration test

Firstly, 0.1 g of dry-weight FB tissue was taken and cut. Then, 1 ml of 50 mM NaOH was added, and the FB organizations were heated at 99 °C. One hundred microliters of 0.1 M (pH 7.4) Tris-HCl was introduced and mixed well after 15 min. Finally, the supernatant was taken after centrifugation and assayed for DNA content on a Nanodrop.

### Statistical analysis

All statistical data were expressed as mean ± SD. GraphPad was employed to analyze all data, while Origin was utilized to plot. Analysis of variance was used to evaluate data involving 3 or more groups. FlowJo software was used to process the flow analysis findings. At *P* < 0.05, statistical significance was established.

## Data Availability

All data supporting the findings of this study are available within the article and its supplementary materials.
