# Supplementary material for: Acoustic Transmitted Decellularized Fish Bladder for Tympanic Membrane Regeneration
Source: Research (Wash D C). 2025 Feb 5;8:0596. doi: 10.34133/research.0596 (PMC11794765; doi:10.34133/research.0596)
Supplement: Supplementary 1 — Figs. S1 to S7 Table S1 [file research.0596.f1.zip › Supplementary Information Table S1.docx]

**Table S1** Target gene primer sequences

| Gene | Forward | Reverse |
| --- | --- | --- |
| TNF-α | AGGCACTCCCCCAAAAGATG | CCACTTGGTGGTTTGTGAGTG |
| IL-1β | TGCCACCTTTTGACAGTGATG | TGTGCTGCTGCGAGATTTGA |
| IL-6 | TAGTCCTTCCTACCCCAATTTCC | TTGGTCCTTAGCCACTCCTTC |
| iNOS | TTCTCAGCCACCTTGGTGAAG | GCTACTCCGTGGAGTGAACA |
| GAPDH | AGGTCGGTGTGAACGGATTTG | TGTAGACCATGTAGTTGAGGTCA |
