# Supplementary figures and images for: Acoustic Transmitted Decellularized Fish Bladder for Tympanic Membrane Regeneration
Source: Research (Wash D C). 2025 Feb 5;8:0596. doi: 10.34133/research.0596 (PMC11794765; doi:10.34133/research.0596)

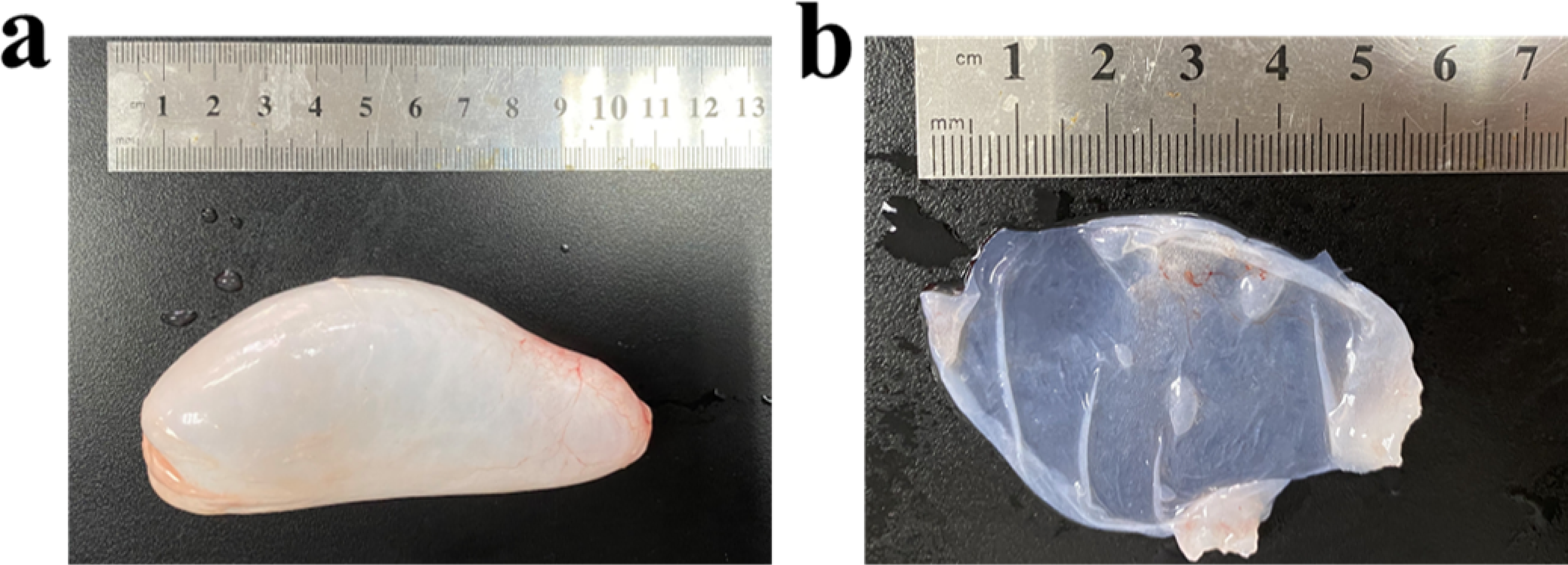

Supplement: Supplementary 1 — Figs. S1 to S7 Table S1 [file research.0596.f1.zip › Figure S1.tif]

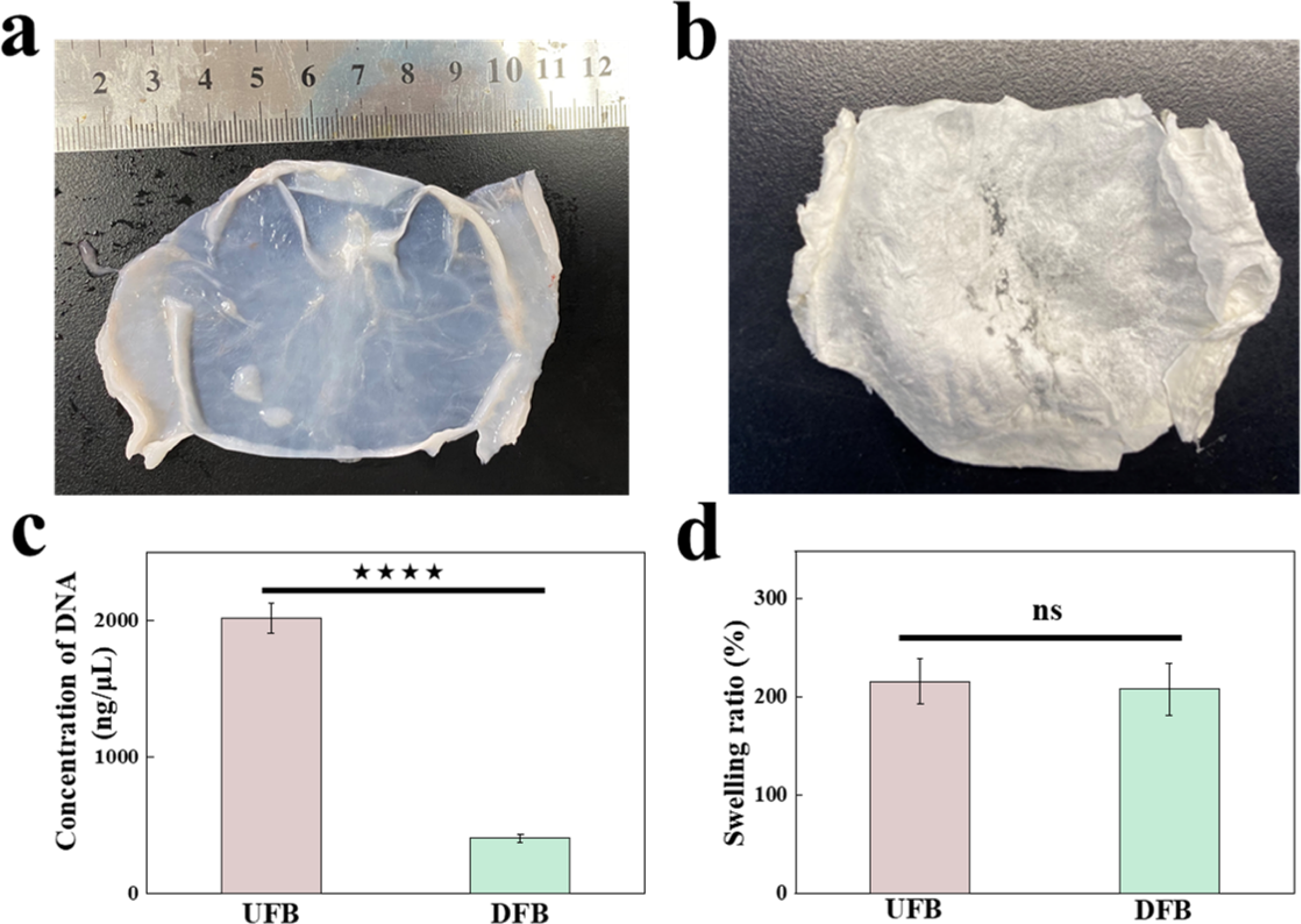

Supplement: Supplementary 1 — Figs. S1 to S7 Table S1 [file research.0596.f1.zip › Figure S2.tif]

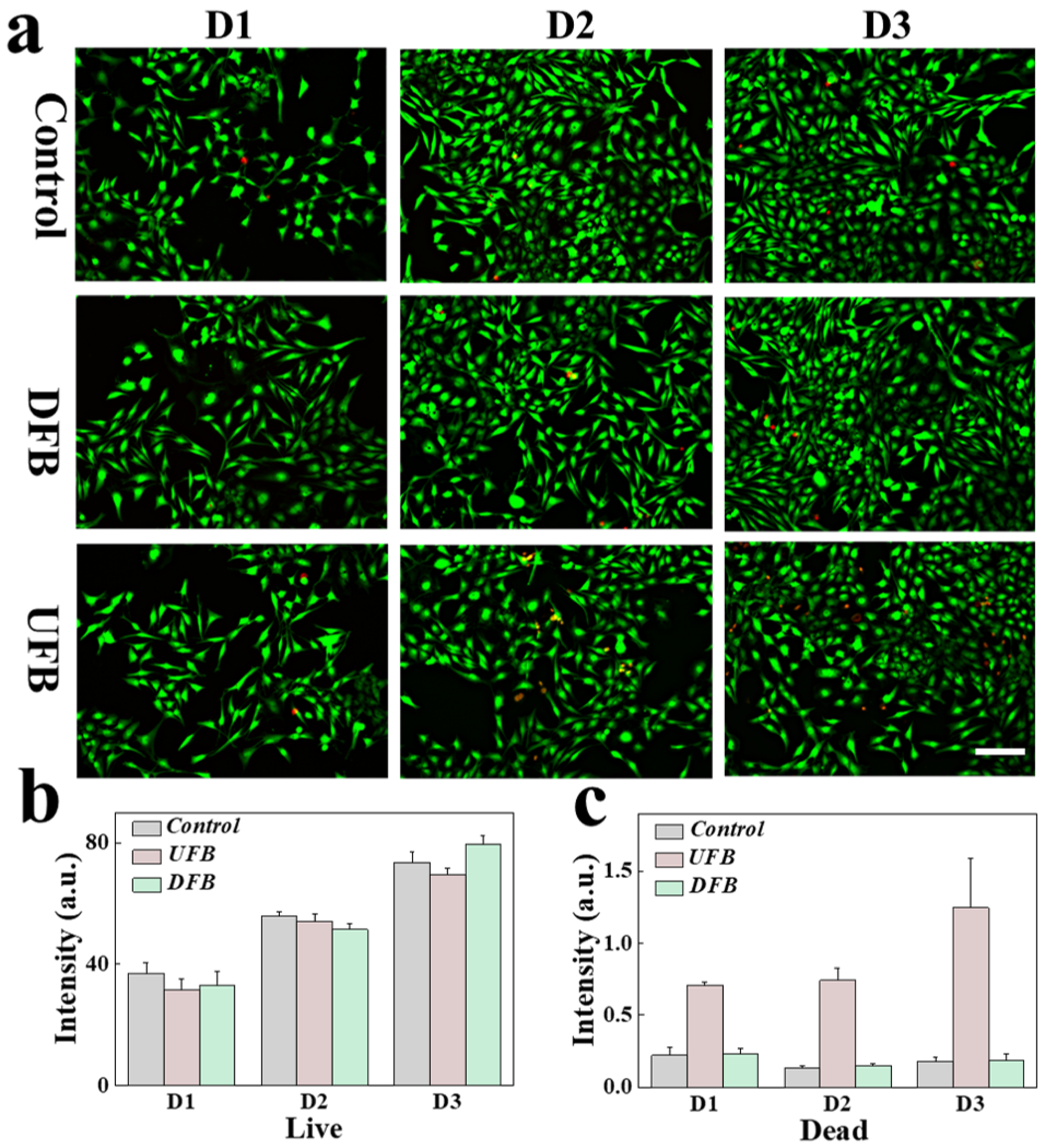

Supplement: Supplementary 1 — Figs. S1 to S7 Table S1 [file research.0596.f1.zip › Figure S3.tif]

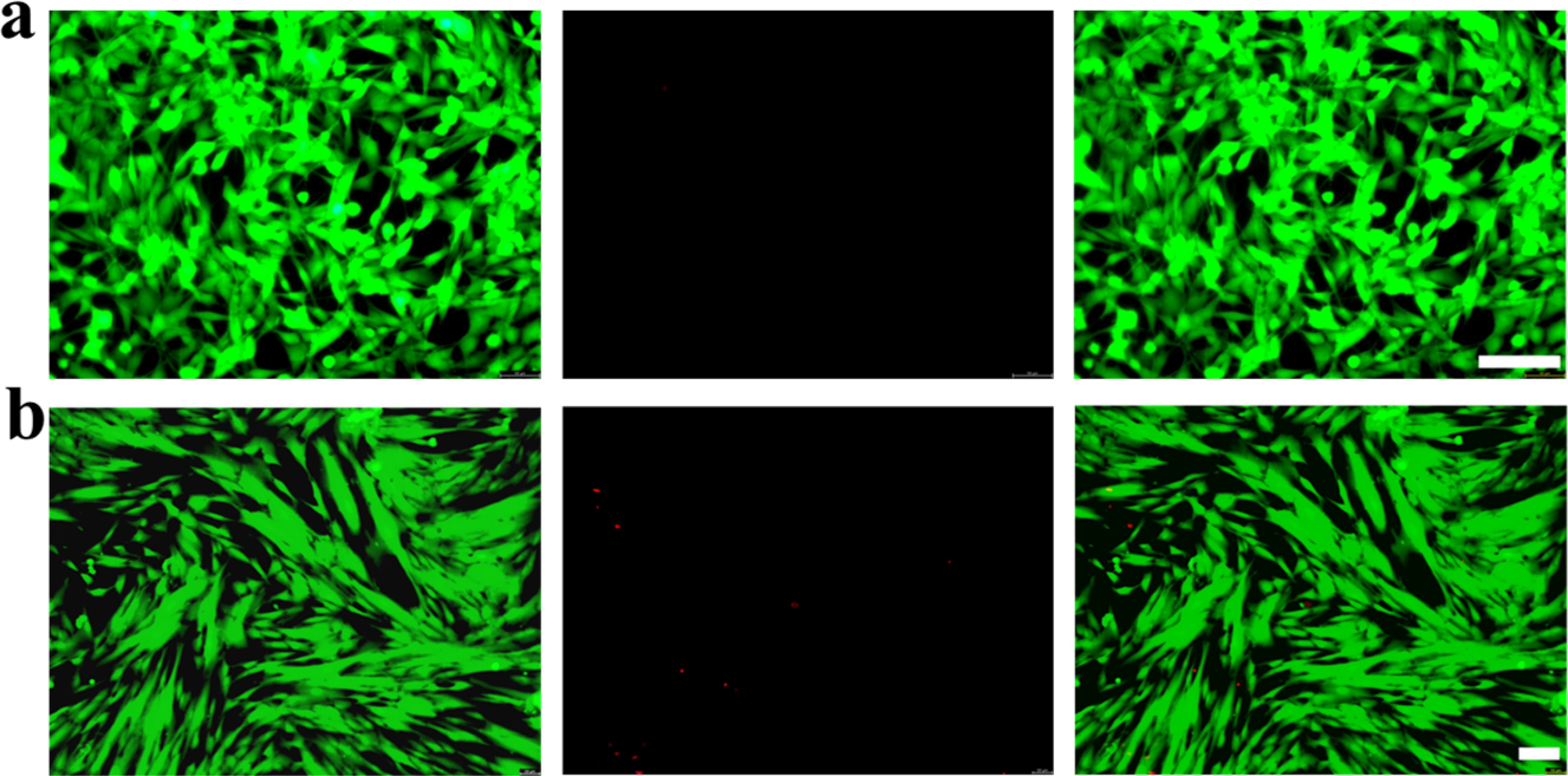

Supplement: Supplementary 1 — Figs. S1 to S7 Table S1 [file research.0596.f1.zip › Figure S4.tif]

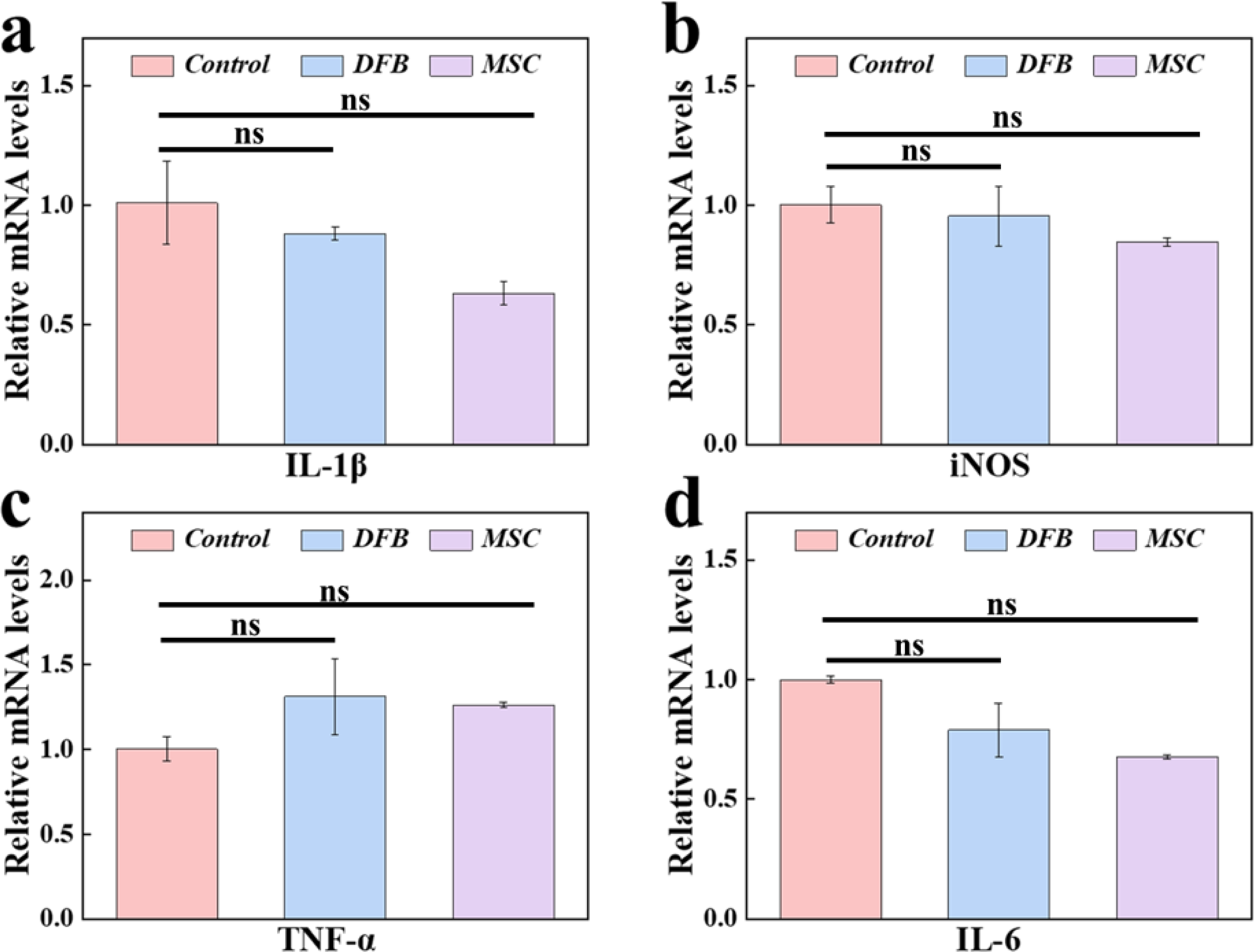

Supplement: Supplementary 1 — Figs. S1 to S7 Table S1 [file research.0596.f1.zip › Figure S6.tif]
